# Precise plan of hybrid treatment for thoracoabdominal aortic aneurysm: Hemodynamics of retrograde reconstruction visceral arteries from the iliac artery

**DOI:** 10.1371/journal.pone.0205679

**Published:** 2018-10-15

**Authors:** Ding Yuan, Jun Wen, Liqing Peng, Jichun Zhao, Tinghui Zheng

**Affiliations:** 1 Department vascular surgery of West China Hospital, Sichuan University, Chengdu, China; 2 School of Civil Engineering and Architecture, Southwest University of Science and Technology, Mianyang, Sichuan,China; 3 Department of Radiology, West China Hospital, Sichuan University, Chengdu, China; 4 Department of Applied Mechanics, Sichuan University, Chengdu, China; Worcester Polytechnic Institute, UNITED STATES

## Abstract

Hybrid visceral-renal debranching procedures with endovascular repair have been proposed as a less invasive alternative to conventional thoracoabdominal aortic aneurysm or dissection (TAAA or TAAD) surgery. Up to now, there has been no information about the hemodynamic effects of retrograde visceral reconstruction (RVR) for Crawford type II/III/IV TAAA patients undergoing hybrid treatment. The aim is to provide insights in the abnormal hemodynamics of RVR from unilateral or bilateral common iliac arteries (CIAs). Idealized three-dimensional AAs with RVR from unilateral CIA or bilateral CIAs were generated and computationally simulated. The results show that RVRs from CIA lead to a dramatic decrease in flow to the visceral organs compared with a healthy AA and that the anastomosis region is most dangerous to graft occlusion and the initiation of an aneurysm. In addition, compared with a quar-furcated graft, the employment of bilateral bi-furcated grafts have better performance in terms of the wall shear stress (WSS) and flow filed but result in less flow to the celiac and mesenteric arteries. This study has revealed the potential risks after an RVR operation, and points out the advantages and disadvantages of different approaches for the visceral reconstruction, which the vascular surgeons are not fully aware of. According to our results, bilateral bi-furcated grafts are recommended to the TAAA patients when the CIAs are unique inflow sites for visceral reconstruction. A precise plan with patient specific for TAAA or TAAD will be designed for better long-term outcome.

## Introduction

There are three operation treatments for TAAA or TAAD: open repair, endovascular repair and hybrid surgery. The hybrid procedure combining open surgical reconstruction of the visceral artery and endovascular aneurysm exclusion was first reported by Quinones et al[1]. Being an alternative approach to high risk patients for open repair of TAAA, the hybrid treatment has the following advantages, 1) avoiding the open burden of thoractomy; 2) avoiding aortic cross-clamping to reduce the visceral ischemia time, which influences the appearance of severe complications such as renal impairment; 3) reducing the risk of neurological complications under hemodynamically stable conditions, such as paraplegia and (or) paraparesis. From 1999 to 2015, about 1000 patients of TAAA were treated by the hybrid method because of the difficulty with open and endovascular repair, as reported around the world[2–5]. Moreover, the numbers of hybrid treatment for TAAA have been growing and some experiences has also been reported in Japan and China[3,6–9].

The majority of patients of hybrid repair underwent RVR from the aortic bifurcation or CIA[2,3] to visceral arteries, in which the inflow site of RVR was selected based on the integrity of the infrarenal abdominal aorta and common iliac artery. For Crawford I/V type TAAA, vascular surgeons may select the infrarenal abdominal aorta or CIA as the retrograde inflow site. However, CIAs are indicated to provide the unique inflow of RVR because of the whole infrarenal aortic artery lesion with Crawford II/III/IV TAAA[10,11]. Our last paper revealed that an non-physiological hemodynamics of RVR from the infrarenal abdominal aortic or CIA will induce some potential risks to the host artery and the visceral organs[12]. However, the hemodynamic effects of RVR for Crawford type II/III/IV TAAA is still unknown.

Moreover, when the normal CIAs are the only inflow sites for RVR, abdominal visceral arteries may be constructed by one quar-furcated graft from unilateral CIA or bi-furcated grafts separately from bilateral CIAs[5]. Vascular surgeons usually perform unilateral CIA or bilateral CIAs based on personal experience, however, which type graft bypassing is more favorable to the organ perfusion? Which one has a better chance of graft patency? What kind of potential risk for the prognosis of this non-physiological RVR? Whether two bi-furcated grafts may guarantee better organ perfusion because of its geometrical symmetry? Whether a quar-furcated graft from unilateral CIA may lead to inadequate perfusion to the organ or the limbs? Up to know, there are no answers or suggestions to all the above questions. Surgeons will precisely select one of RVR methods in patients of TAAA or TAAD with hybrid treatment if above all questions were clearly indicated.

In recent years, numerical investigations have been used increasingly by researchers seeking to understand vascular hemodynamics. Many researchers have shown that computational fluid dynamics (CFD) can faithfully capture the physics of various vascular diseases including abdominal aortic aneurysm, cerebral aneurysm, aortic dissection, artery stenosis etc[13–15]. In addition, the hemodynamics of vascular diseases following endovascular treatment were also widely investigated using CFD[16]. Good consistency between the actual hemodynamics and numerical estimations has been acknowledged in the biomechanics and medical field, and it is widely accepted that CFD results are quite useful clinically and have the potential to provide practical insights intro vascular surgery.

Accordingly, this manuscript focuses on the hemodynamic influence of RVR on the aorta with Crawford II/III/IV TAAA using the CFD method described in our last paper[12]. This work and our last paper are mutually complementary and combine to reveal the hemodynamics of RVR with hybrid treatment for all types of TAAA, In addition, this study also aims to make a comparison between the employment of two bi-furcated grafts and one quar-furcated graft for RVR in terms of their hemodynamic performances so as to assess the ongoing performance of the visceral grafts and anastomotic sites. Based on our CFD results, a better RVR of hybrid surgery will reasonably selected and hybrid treatment will be precisely performed for better outcome for TAAA patients.

## Model and methods

### Model geometry

**Fig 1:**
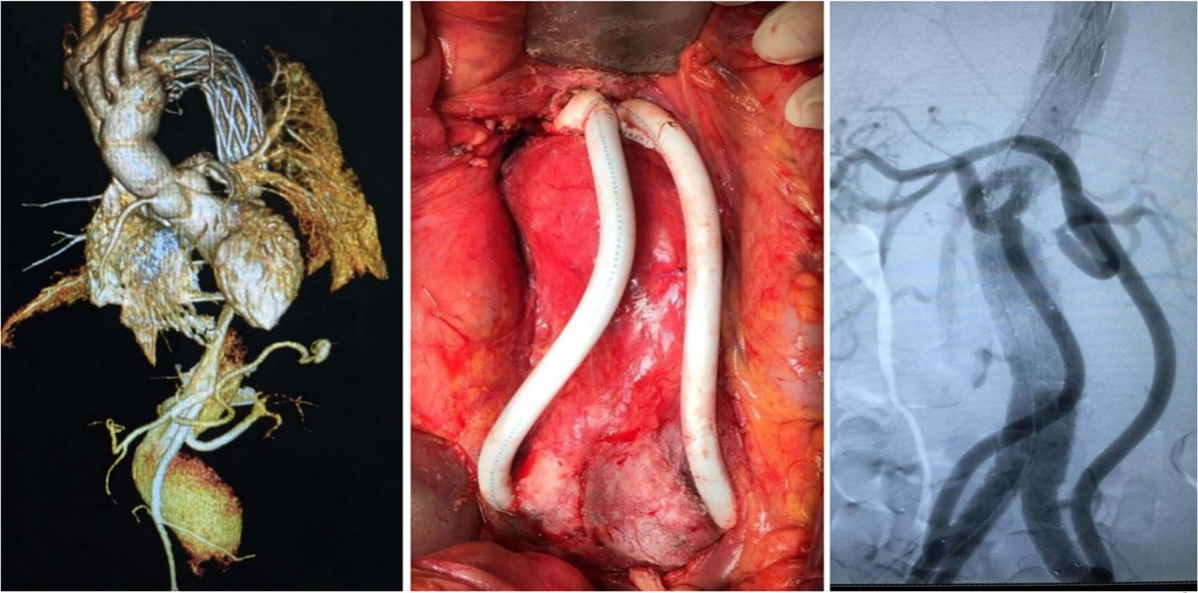
Hybrid treatment for Crawford type III TAAA with Marfan syndrome. Left: the preoperative CTA image of TAAA; Middle: the retrograde revascularization from bilateral CIA; Right: The angiography of DSA for the RVR with bilateral CIA following hybrid treatment.

The simple procedures of RVR with unilateral and bilateral CIA are:

1. For unilateral CIA, one quar-furcated graft was constructed, and the main body side of a quar-furcated graft was anastomosed end-to-side to a unilateral CIA with a 5–0 Prolene suture to serve as the visceral inflow blood source. The four limbs of the graft were respectively anastomosed, end-to-side, to the right renal artery, superior mesenteric artery (SMA), celiac artery (CA) and left renal artery (LRA) with a 6–0 Prolene suture[3].
2. For bilateral CIAs, two bifurcated grafts were chosen, and the main body side of one bifurcated graft was anastomosed end-to-side to a right CIA that served as the visceral inflow blood source. The two limbs of the graft were respectively anastomosed end-to-side to right renal artery (RRA) and CA. The other graft was constructed from a left CIA to SMA and LRA (Fig 1).
3. The bifurcated vessel prostheses (Gore-TEX) of 16mm×8mm were actually selected for the bypass graft of RVR based on the experience in the West China Hospital. The vessel prostheses (Gore-TEX) of 16mm×8mm were more suitable to Asian populations than the prostheses of 18mm×9mm because of the smaller diameter of arteries in Asian populations[17]. Further, two 8mm grafts were respectively sewn side-to-end to the main body of the 16×8mm Y-shape bifurcated graft, thus allowing the tetrachotomous graft to be constructed. Finally, the inflow diameter of the bypass graft model was 16mm and the outflow diameter was 8mm.

**Fig 2:**
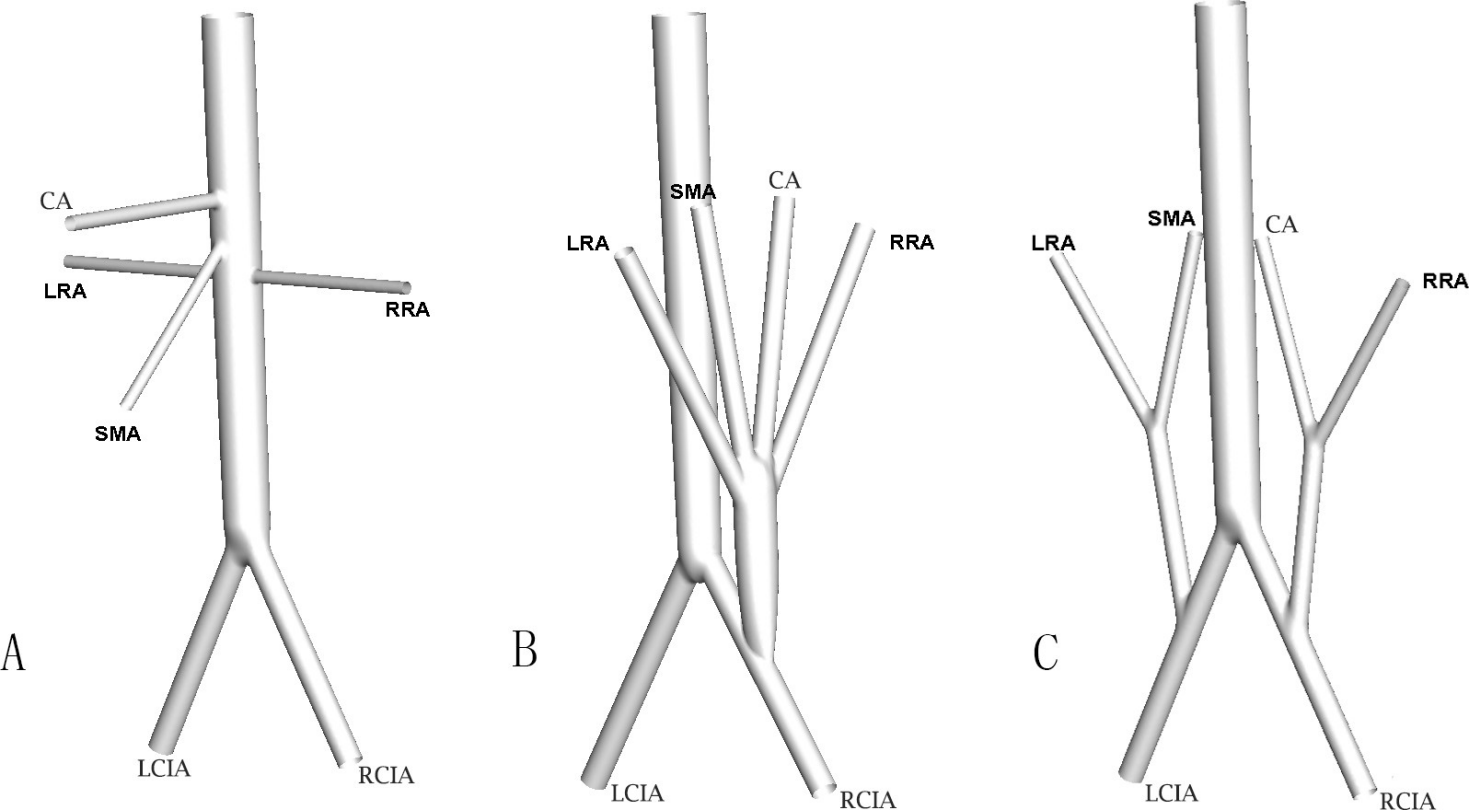
Sketches of model geometry. A) Structure of normal AA; B) RVR using unilateral quar-furcated graft; C) RVR using bilateral bi-furcated grafts. CA-celiac artery, LRA-left renal artery, RRA-right renal artery, SMA- superior mesenteric artery, LCIA-left common iliac artery, RCIA-right common iliac artery.

The model geometry was built using software Pro/E 5.0 (PTC Inc., Massachusetts, USA). Based on the postoperative computer tomography angiography (CTA) or DSA of patient with TAAA[3], the normal abdominal aortas (AA) [18] and AA with visceral grafts connected to the iliac arteries shown were constructed (Fig 2).The aorta tapered uniformly from a circular cross section with a diameter of 22mm at the supraceliac aorta to a circular cross section with a diameter of 16mm at the aortic bifurcation. The take-off angles of both iliac arteries at the bifurcation were 30°. The idealized AAs with visceral branches were constructed referring to the average data of other healthy Chinese cases[17,18] (Fig 2A). To simplify the modeling, the decimal values were omitted for the numerical values of the diameters. When a quar-furcated graft was connected to the left common iliac artery (LCIA), the diameters of the bypass and debranching grafts were 16 mm and 8mm respectively (Fig 2B). When two bi-furcated grafts were connected, the diameters of the bypass and visceral grafts were all 8mm (Fig 2C). To make a fair comparison, the anastomosis angles between the host artery and the graft were all set to be 45°.

### Governing equations

In the current study, the blood was assumed to be an incompressible, laminar, steady, homogenous and Newtonian fluid, and the corresponding governing equations are given as

$$\rho (\vec{u}\cdot \nabla )\vec{u}+\nabla p-\mu \Delta \vec{u}=0$$ (1)

$$\nabla \cdot \vec{u}=0$$ (2)

Where $\vec{u}$ and *p* represent, respectively, the fluid velocity vector and the pressure. *ρ* and *μ* are the density of 1050*kg*/*m*^3^ and dynamic viscosity of 3.5×10^−3^ *kg*/*m*⋅*s*.

### Boundary conditions

First, the entrance boundary of the supraceliac artery was defined as having a constant-velocity inlet of 0.18*m/s*. Due to the clinical fact that the perfusion pressure of the visceral arteries are consistent if no artery stenosis happens, namely, the perfusion pressure for the visceral organs remain unchanged before and after the operation. So we first calculated the corresponding pressure drop from the supraceliac artery to each visceral organ in terms of the flowrate ratio for a healthy AA model, then we applied the same perfusion pressure to the corresponding visceral organ for the RVRs. In another words, assuming that the pressure at the supraceliac artery was 100 mmHg, the outlet pressure at each visceral artery was then defined taking into account the corresponding pressure drop for a healthy AA model[12]. The iliac outlet pressure was selected so that the entire flow rate was the same as that for the healthy one.

The graft and vessel wall were assumed to be rigid and nonslip.

### Numerical simulation

**Fig 3:**
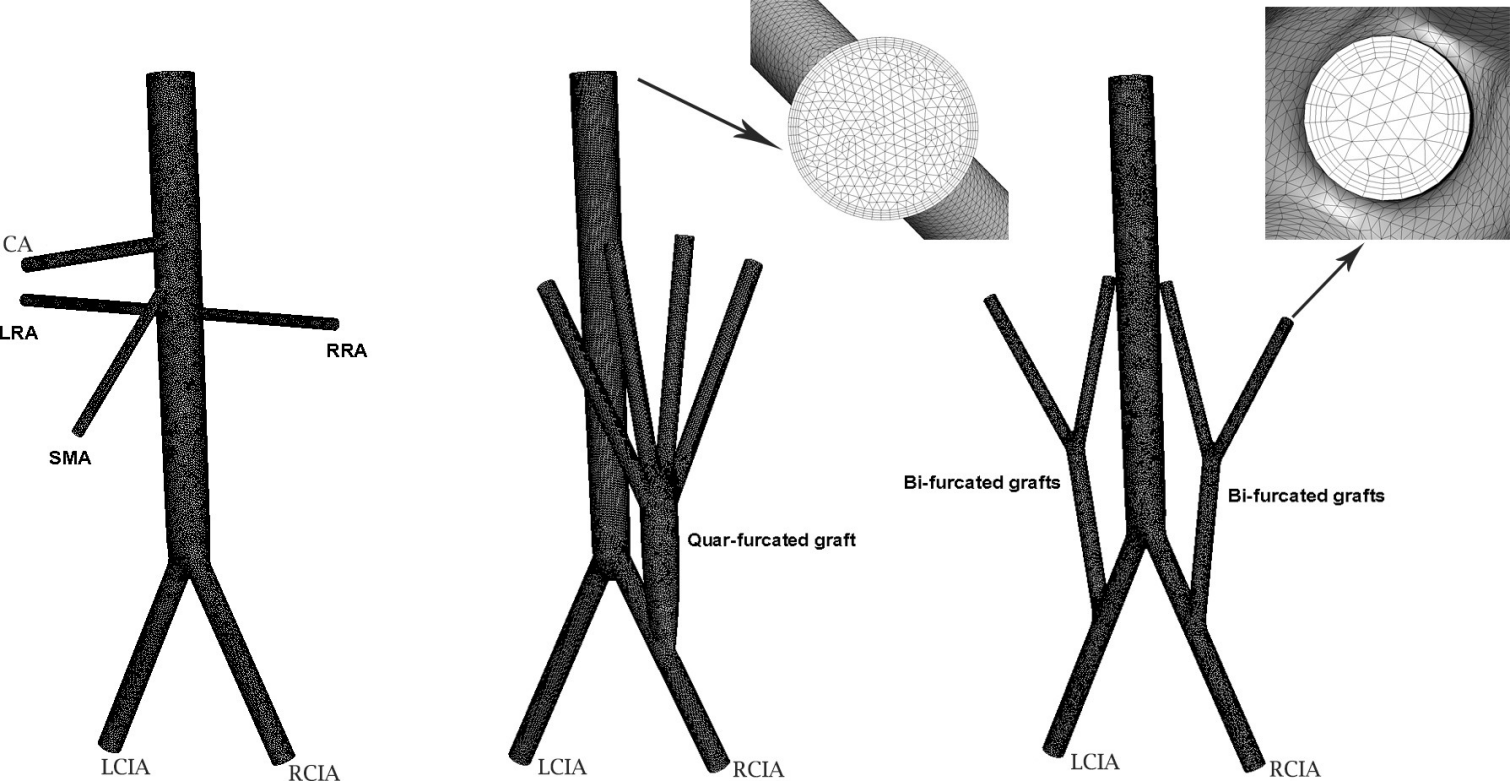
Mesh generations of AA with RVR; A) Structure of normal AA; B) AA with quar-furcated bypass graft; C) AA with bi-furcated bypass graft; D) Zoom-in View of boundary layer.

Meshes in the region of the aorta below the renal arteries, the aortic bifurcation and along the branch vessels were refined. In addition, the boundary layer was resolved by placing the first four grid nodes at approximately 15, 17, 19 and 21μm away from the wall, and we supplemented the zoom-in view of the boundary layer mesh in Fig 3.

The flow visualization and analysis were completed by the commercial CFD software Ansys FLUENT 12.0 which was based on the finite volume method. The default segregate implicit 3D solver was applied. Discretization of the equations involved a second order upwind differencing scheme, SIMPLE was adopted for the pressure velocity correction and the residual error convergence threshold was set as 1e-5.

**Fig 4:**
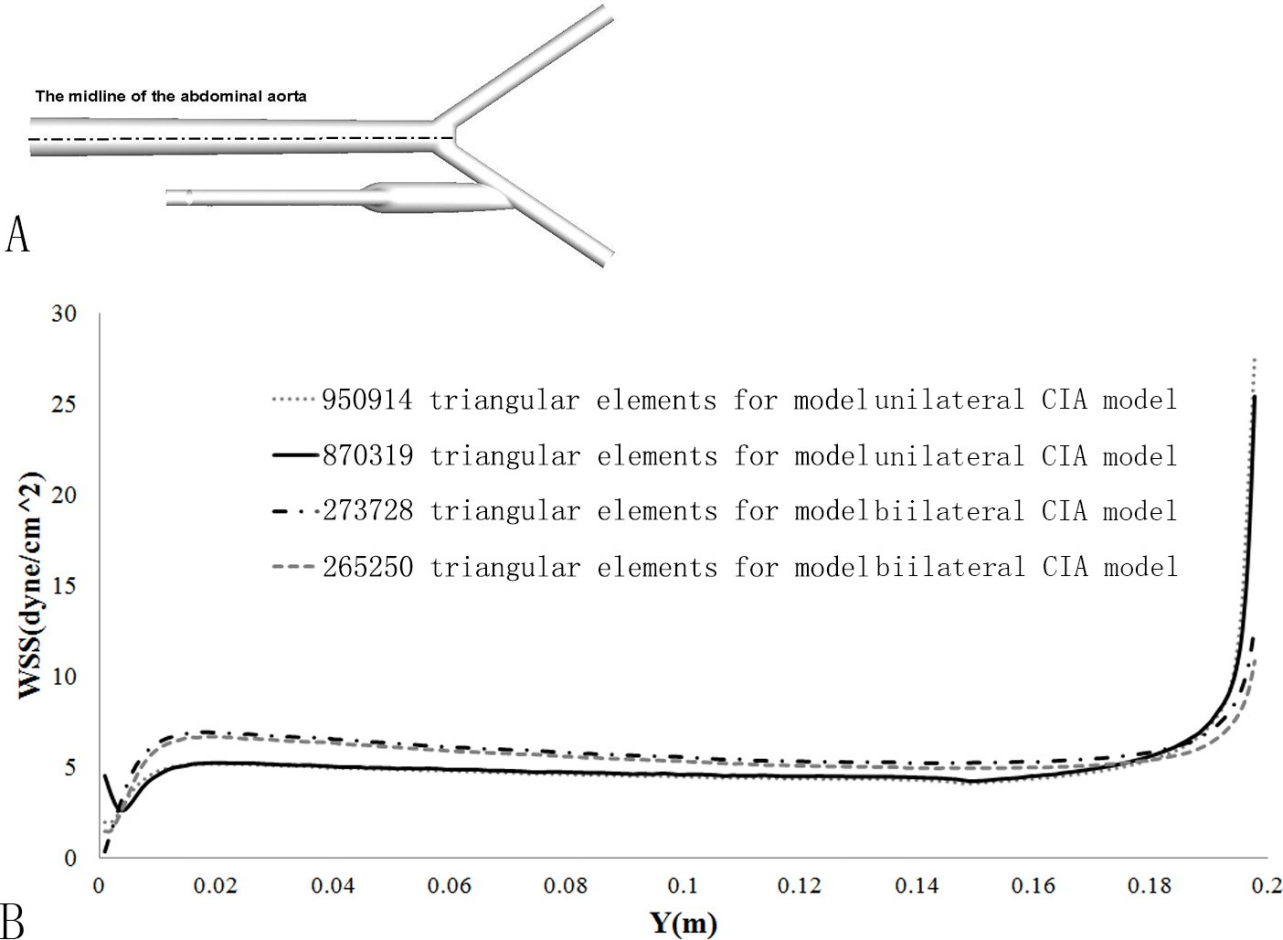
3D Grid independence test. The WSS distributions along the aorta centerline were presented at two different meshes.

To establish a grid-independent solution, a fine mesh consisting of approximately 950914 cells and a coarse mesh of 870319 cells for the unilateral CIA model while two meshes of 273728 and 265250 cells, respectively, for the bilateral CIA model were tested. As is shown in the WSS distribution along the aorta centerline, the maximum relative errors of the WSS magnitudes were 2.3% and 3.5%, respectively (Fig 4). Therefore, the course grids were considered satisfactory and adopted for the following investigation.

## Results

**Fig 5:**
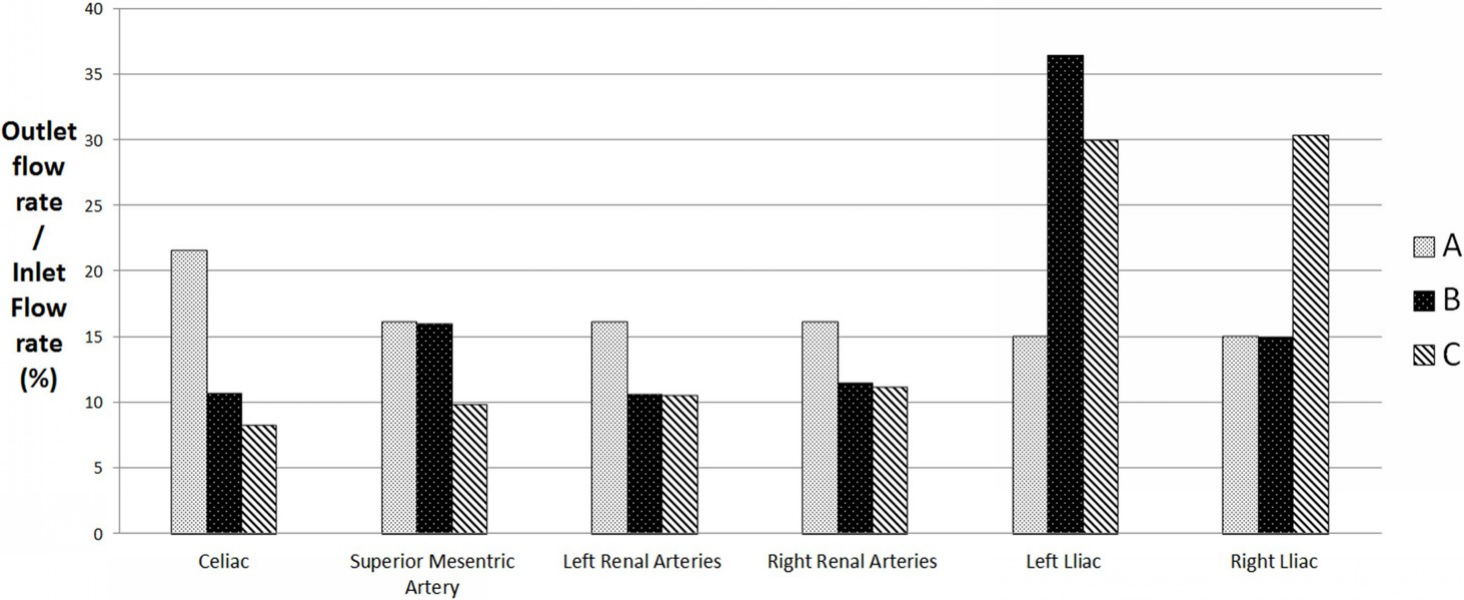
Histograms of the flow split for each branch artery A) healthy abdominal aorta; B) RVR using unilateral quar-furcated graft; C) RVR using bilateral bi-furcated grafts.

Fig 5 presents the histograms of flow split to each branch artery of the AA for two cases. For a healthy AA, 70% of the blood that enters the aorta was prescribed to its visceral arteries and the remaining 30% flowed down the infrarenal segment through the bifurcation into the iliac arteries[18]. When a quar-furcated graft was connected to the left common iliac artery, 48.56% blood entering the supraceliac aorta was assigned to the digestive and renal arties, and the volume flowing down to the iliac arteries accounted for 51.44% of the total inflow to the AA. The flow volume to the right common iliac artery (RCIA) is two times more than that of the LCIA. When bi-furcated grafts were connected bilaterally, 39.65% of the total blood was assigned to the visceral organs, and each iliac artery received the remaining blood flow. Specifically, for a quar-furcated graft or bi-furcated graft, the percentages of blood flowing to the celiac artery were 10.67% and 8.19%, to the SMA were 15.96% and 9.81%, to the left renal artery were 10.64% and 10.49%, to the right renal artery were 11.44% and 11.17%, to the LCIA were 36.48% and 30%, and to the RCIA were 15% and 30.35% respectively (Fig 5). As it revealed, that the employment of the two bi-furcated visceral grafts brought almost no volume change to the renal arteries but redistributed the flow to the other organs and iliac arteries.

**Fig 6:**
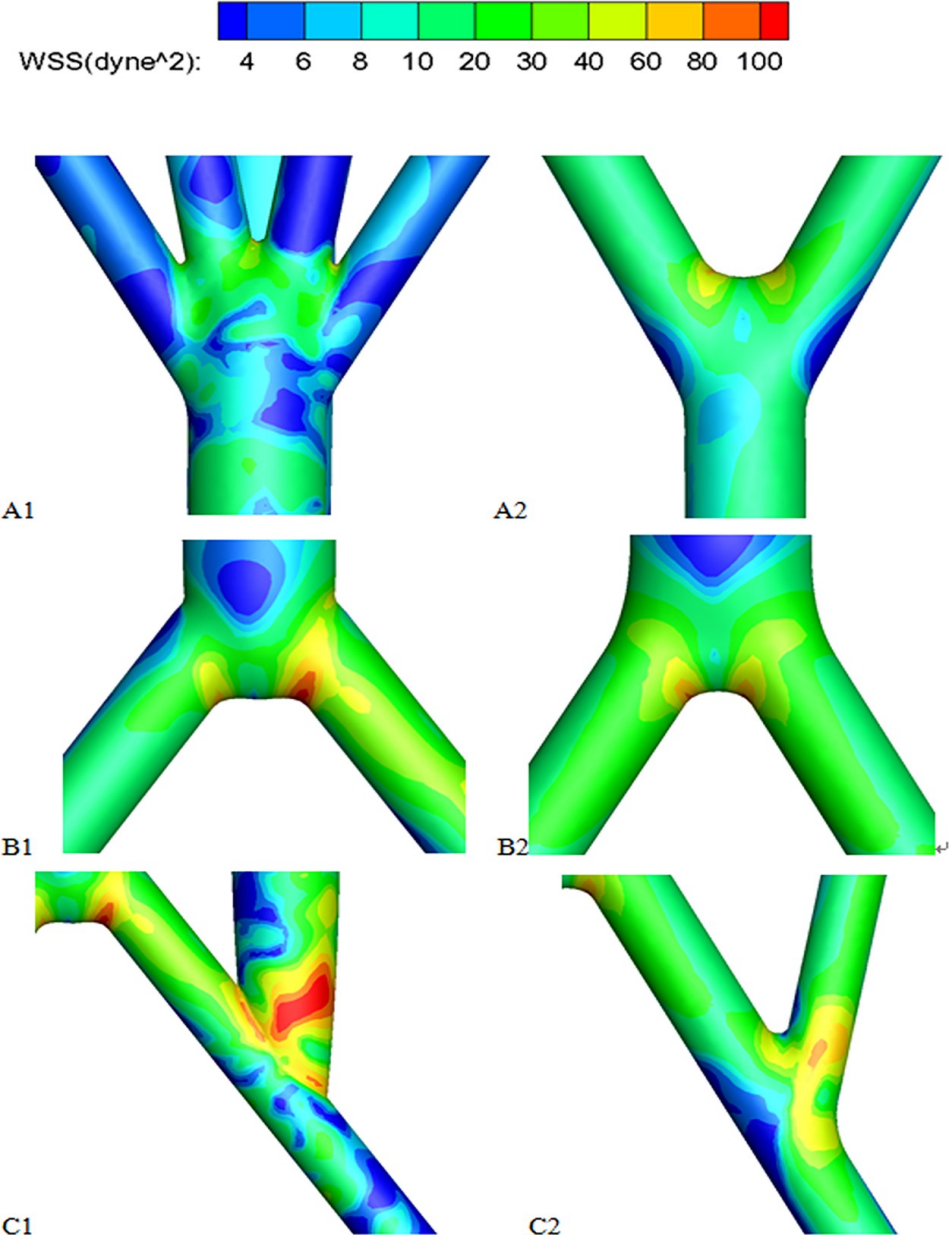
The Zoom-in WSS contour maps. A1, A2)the graft bifurcation; B1, B2) the aorta bifurcation; C1, C2) the anastomosis region. A1, B1, C1 represent the local regions of the RVR with unilateral quar-furcated graft; A2, B2, C2 represent the local regions of the RVR with bilateral bi-furcated grafts.

The WSS contour maps of the graft bifurcation, the aorta bifurcation and the anastomosis region are shown in Fig 6. Physiological WSS values typically range from 10 to 50 dyne/cm^2^ in the arteries[19], and it was found that the platelets became activated when exposed to shearing forces more than 100 dyne/cm^2^ for only very brief durations[20]. Therefore, the extremely low WSSs, smaller than 10 dyne/cm^2^, are marked in blue while extremely high WSSs, larger than 100 dyne/cm^2^, are marked in red in the Figures. There are three prominent features needing to be addressed. First, compared to the bi-furcated graft, the four-furcated graft resulted in more areas with low WSS. For example, most areas of the four-bifurcated visceral grafts were plagued with low WSS while only part of the side walls of the bi-furcated graft were affected. In addition, although for both cases, there were low WSS regions on the artery floor at the anastomosis, only the toe and swing section of the quar-furcated graft suffered from low WSS. Secondly, when a bi-furcated graft was set up, the WSS on its iliac arteries were in the normal range but it was not true when a quar-furcated graft was adopted. Finally, when a unilateral quar-furcated graft was used, extremely high WSS appeared on the aorta bifurcation and anastomosis region of the inflow site (Fig 6).

**Fig 7:**
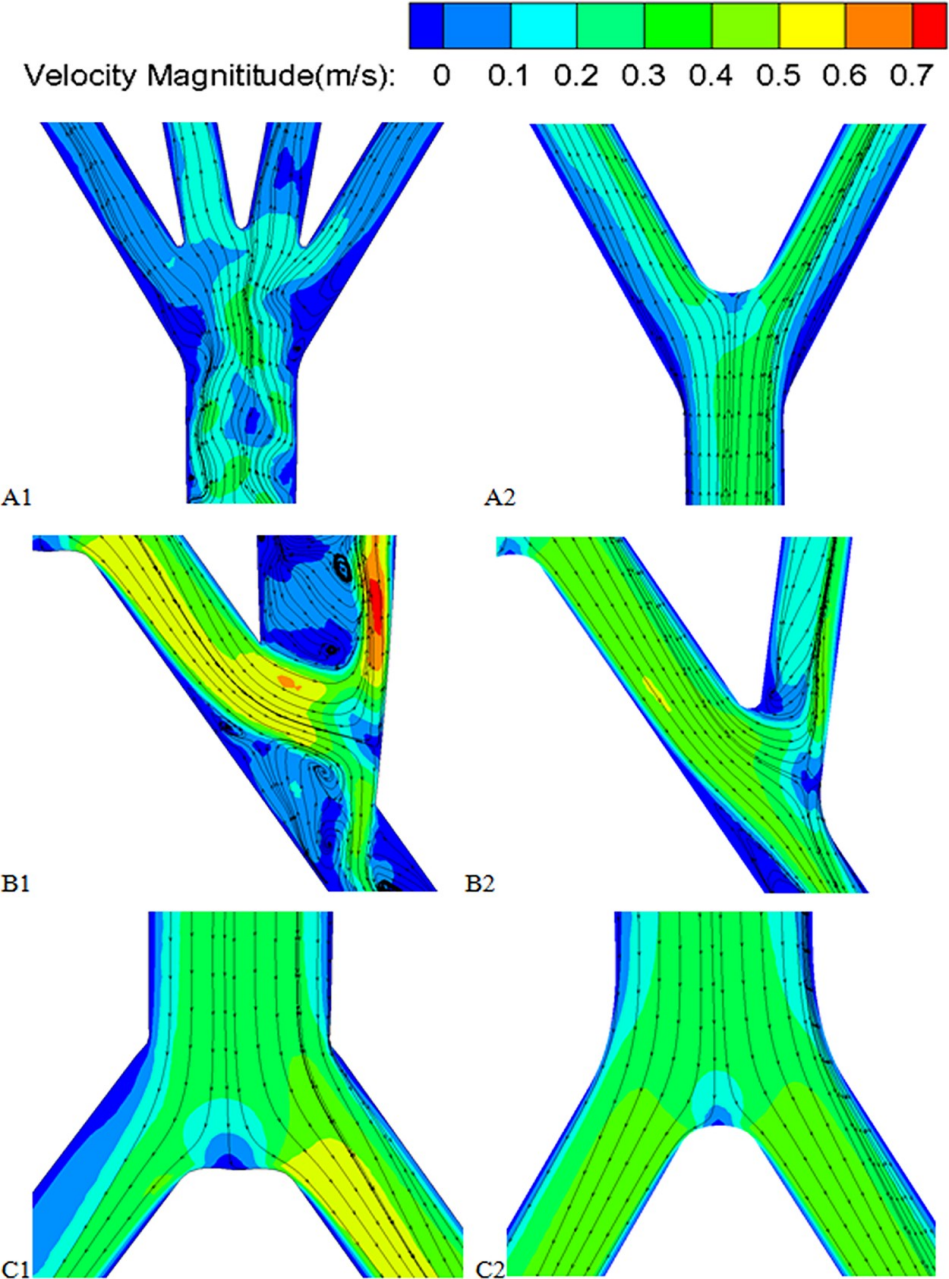
The streamlines superimposed the contour maps of velocity magnitude in the symmetry planes. A1, A2) the graft bifurcation; B1,B2) the aorta bifurcation; C1,C2) the anastomosis region. A1,B1,C1 represent the local regions of the RVR with unilateral quar-furcated graft; A2,B2,C2 represent the local regions of the RVR with bilateral bi-furcated grafts.

Fig 7 shows the streamlines superimposed on the contour maps of the velocity magnitude in the planes of symmetry. For both cases, when fluid was flowing from the host artery to the graft, the sudden geometric change shifted the fluid towards the outer wall of the graft under the action of circumferential forces, and flow recirculation zones appeared near the inner wall, the toe region and host artery bed. It is obvious that compared with the bi-furcated graft case, the quar-furcated graft had a more disturbed and chaotic flow pattern, not only in the anastomosis region, but at the graft bifurcation.

**Fig 8:**
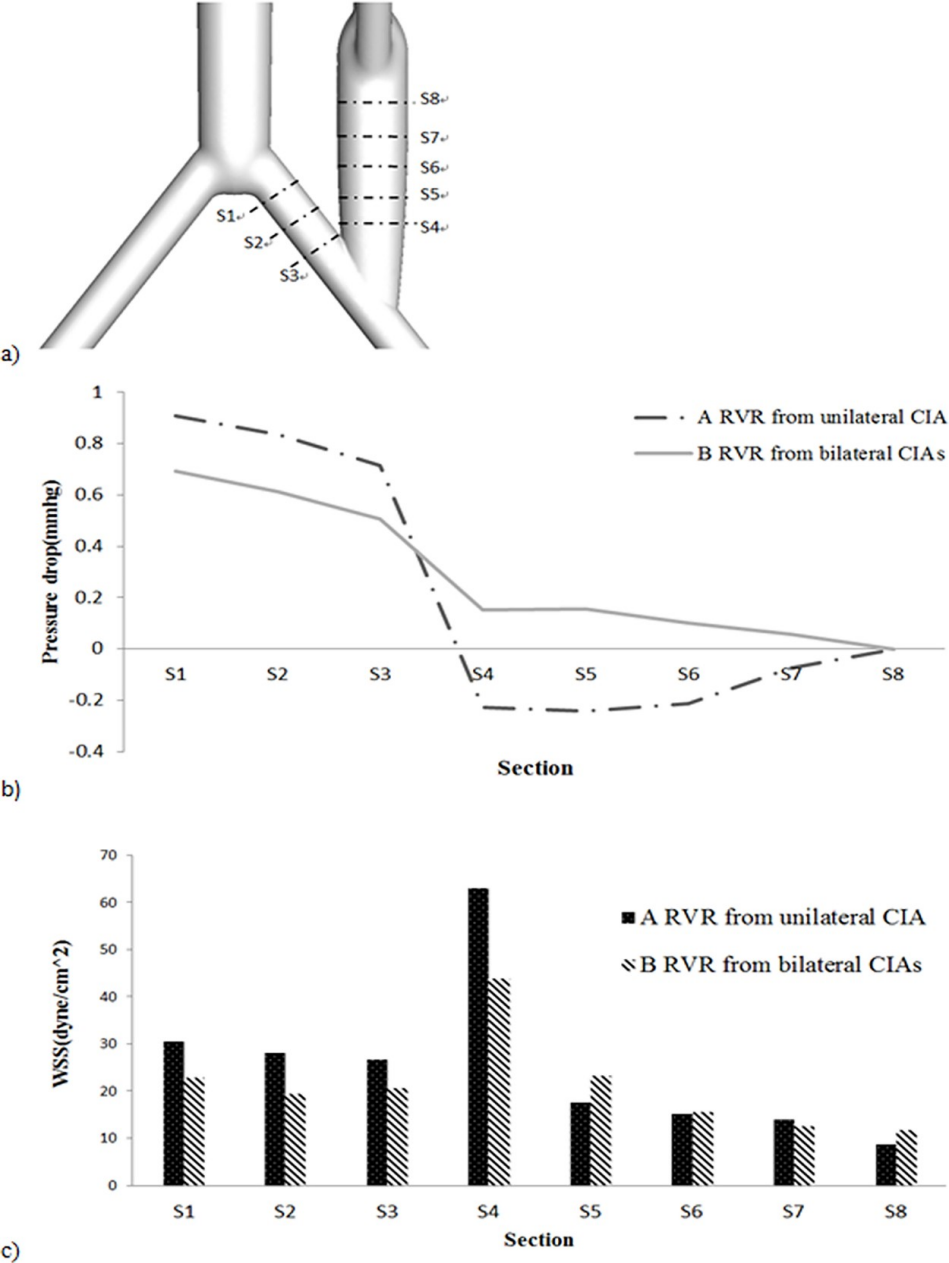
To have a better view of the effect of graft type on the hemodynamic parameters, 8 slices of the configurations were chosen for the calculation of the area-averaged WSS and pressure drop. a) the sketch of the 8 slices. b) the area-averaged pressure drop. c) area-averaged WSS.

To have a better view of the effect of the graft type on the hemodynamic parameters, 8 slices of the configurations, as shown in Fig 8, were chosen for the calculation of the area-averaged WSS and pressure drop. For both cases, the WSS experienced a sharp increase from S3 section to S4 section at the artery-graft anastomosis. A unilateral quar-furcated graft gave rise to a more fluctuating WSS, and the averaged WSS of the S4 section was more than 50 dyne/cm^2^, while that of the S8 section less than 10 dyne/cm^2^, both crossing the safety line of the normal WSS range in an artery. In contrast, the bilateral bi-furcated grafts resulted in more evenly distributed WSSs with values were all in the normal range (Fig 8C). In addition, the main pressure drop was situated over the anastomosis (S3-S4) for both cases. For the bilateral bi-furcated graft, the pressure decreased linearly in the iliac artery (cross section S1-S3) and the graft (S5-S8). However, when the unilateral quar-furcated graft was applied, a negative pressure gradient occurred between the S6 and S8 sections, inferring a possible flow reversal there (Fig 8B).

## Discussion

This paper aims to investigate the general features and phenomena of the hemodynamics in an aorta with RVR for patients with Crawford II/III/IV TAAA. We adopted idealized 3D aorta geometries with a quar-furcated graft from unilateral CIA or bi-furcated grafts separately from bilateral CIA for the numerical investigation. The computational results revealed that: 1) No matter whether a unilateral quar-furcated graft or a bilateral bi-furcated graft, RVRs from the CIAs result in a dramatic decrease in the flow split to the visceral organs, compared with a healthy AA. The unilateral grafts have a 30% decrease while the bilateral grafts have a 43% decrease, which represents a potential risk of inadequate perfusion after the RVR operation. 2) No matter the method of reconstruction from CIAs, the anastomosis regions are plagued with low and unevenly distributed WSS, flow recirculation and stagnation and flow skewness. In addition, comparison between the two different reconstruction from the CIAs showed that the bifurcated grafts from bilateral CIAs have the following benefits: 1) the employment of bilateral bi-furcated grafts for visceral rerouting significantly eliminated the low WSS region, WSS gradient change and flow recirculation, not only in the anastomosis region but in the graft sections, compared to a unilateral quar-furcated graft, 2) When a unilateral quar-furcated graft is set up, parts of its anastomosis region are exposed to extremely high WSS (more than 100 dyne/cm^2^), which is not the case when bilateral bi-furcated grafts are deployed. 3) The symmetrical bilateral bi-furcated graft brought symmetrical flow splits to the iliac arteries, but extreme low flow volume to the host iliac artery was observed when a quar-furcated graft used. However, the bilateral bi-furcated grafts resulted in a more significant decrease in flow to the visceral organs, especially a more remarkable decrease in flow to the celiac and superior mesenteric arteries.

It is well accepted that hemodynamics is one of the most important factors contributing to aneurysm pathophysiology and plays a fundamental role in the mechanisms of initiation, growth, and rupture[21,22]. Especially, WSS, the frictional drag force per unit area, is predominantly used to identify “unfavorable” hemodynamic conditions because it can be sensed directly as a force on an endothelial cell at the luminal surface[18,23]. Generally, a physiological WSS value ranging from 10 to 50 dyne/cm^2^ is required to keep the normal cell morphology in the human blood vessels. Low WSS is believed to trigger an inflammatory-cell-mediated pathway, while high WSS triggers a mural-cell-mediated pathway, which of both are associated with the growth and rupture of aneurysms[13]. In addition, in the initiation of thrombosis, flow conditions near the vessel wall regulate how quickly reactive components are delivered to the injured site and how rapidly the reaction products are disseminated[19]. For example, platelet activation, a precursor for thrombosis, has been shown to be a function of elevated shear stresses which are generally confined to the vessel wall, and the shear layer between the fluid jet and the recirculation zone in the separated region. Moreover, hypertension, a reflection of increased hoop stress, leads to stiff, thick blood vessels, which can restrict the blood flow and do not respond to the normal physiologic fluctuations in the blood flow. It has been identified as an independent risk factor of abdominal aortic or cerebral aneurysm growth and rupture[24].

Accordingly, in terms of hemodynamics, we believe that for the Crawford II/III/IV TAAA patients who go through RVR from CIAs, a big concern is the inadequate perfusion to the visceral organs, and regular follow-up monitoring of the function of the visceral organs are highly recommended. In addition, the anastomosis region is highly dangerous for the initiation and the progression of stenosis and aneurysm, and graft design including graft size, and anastomosis angle may be carried out later on to reduce the risk of the related complications of the stent graft or blood vessel prostheses or the potential dilation of CIA. Moreover, when the CIA is the only candidate for the RVR of patients with Crawford II/III/IV TAAA, we believe that a bilateral bi-furcated graft has better performance in suppressing the initiation and development of graft occlusion, and in decreasing the vessel wall dilation and reducing artery aneurysms developing at the host iliac artery which helps to prolong the graft patency.

As a preliminary investigation, there are some possible limitations in our investigation:

1. The simulations were carried out under steady flow conditions, which are different from in vivo pulsatile flow conditions. Flow pulsatility may play important protective roles in respect of atherosclerosis and aneurysm, and a large amplitude pulsation may increase the values of the minima in the WSS and velocity, thus enhancing the ‘sweeping’ motion in the blood vessels[25]. But as a common assumption in the hemodynamic investigation of blood flow, steady-state simulations qualitatively capture the average effect of the flow and can also reflect, to a certain extent, the essence of the investigated problem[26].
2. Wall compliance is neglected in this study, which is not true in vivo; however, previous studies have indicate that the wall elasticity may be of considerable significance in, for example, transport mechanisms, but of somewhat lesser importance as far as the gross features of the flow are concerned[27].
3. The assumptions of constant viscosity ignores the non-Newtonian property of blood rheology. However, previous studies have indicated that as far as the hemodynamic parameters such as WSS, flow field and pressure drop and so on are concerned, the results predicted by computations using non-Newtonian properties are quantitatively and qualitatively close to those assuming a Newtonian fluid, hence, we found it reasonable to make the assumption of Newtonian blood in this study[28].

## Conclusions

Vascular surgeons are more concerned about surgical techniques for TAAA treatment while ignoring different hemodynamic effects from different reconstructions. This study firstly indicated hemodynamic characteristic with RVR from CIAs, and in spite of the limitations mentioned above, revealed the potential risks, including the visceral organ ischemia, graft stenosis, thrombosis and anastomosis aneurysm after an RVR operation, and pointed out the advantages and disadvantages of different bypass procedures. Surgeons will plan a precise perform with patient specific for TAAA or TAAD base on this results. Future clinical or animal studies are needed to explore how the decreased flowrate to the visceral organs may affect their function and confirmed the threshold of visceral or limb ischemia. In addition, at this time, the choice of a bypass graft for RVR is limited and we expect that, in the near future, graft design including graft size and anastomosis angle etc. may help to improve the graft patency and decrease postoperative complications. Most importantly, this study again emphasizes the importance and necessity of regular postoperative follow-up to patients for the better recovery.
